# 4-[(2-Chloro­ethyl)amino]quinolinium chloride monohydrate

**DOI:** 10.1107/S160053680904834X

**Published:** 2009-11-21

**Authors:** Marcus V. N. de Souza, Edward R. T. Tiekink, James L. Wardell, Solange M. S. V. Wardell

**Affiliations:** a Instituto de Tecnologia em Farmacos, Fundação Oswaldo Cruz (FIOCRUZ), FarManguinhos, Rua Sizenando Nabuco, 100, Manguinhos, 21041-250 Rio de Janeiro, RJ, Brazil; b Department of Chemistry, University of Malaya, 50603 Kuala Lumpur, Malaysia; c Centro de Desenvolvimento Tecnológico em Saúde (CDTS), Fundação Oswaldo Cruz (FIOCRUZ), Casa Amarela, Campus de Manguinhos, Av. Brasil 4365, 21040-900 Rio de Janeiro, RJ, Brazil; d CHEMSOL, 1 Harcourt Road, Aberdeen AB15 5NY, Scotland

## Abstract

In the title salt hydrate, C_11_H_12_ClN_2_^+^·Cl^−^·H_2_O, the quinolin­ium core is essentially planar (r.m.s. deviation = 0.027 Å) with the chloro­ethyl side chain being almost orthogonal to the core [C—N—C—C torsion angle = −80.0 (3)°]. In the crystal packing, the water mol­ecule bridges three species, forming donor inter­actions to two chloride anions and accepting a hydrogen bond from the quinolinium H atom. The chloride anion accepts a hydrogen bond from the amine N atom with the result that a two-dimensional supra­molecular array is formed in the *ac* plane. A C—H⋯Cl interaction also occurs.

## Related literature

For background to malaria, see: Snow *et al.* (1999 ▶); Breman (2001 ▶); World Health Organization (1999 ▶). For background information on the pharmacological activity of quinoline derivatives, see: Elslager *et al.* (1969 ▶); Font *et al.* (1997 ▶); Kaminsky & Meltzer (1968 ▶); Musiol *et al.* (2006 ▶); Nakamura *et al.* (1999 ▶); Palmer *et al.* (1993 ▶); Ridley (2002 ▶); Sloboda *et al.* (1991 ▶); Tanenbaum & Tuffanelli (1980 ▶); Warshakoon *et al.* (2006 ▶). For recent studies on quinoline-based anti-malarials, see: Andrade *et al.* (2007 ▶); Cunico *et al.* (2006 ▶); da Silva *et al.* (2003 ▶); de Souza *et al.* (2005 ▶). For a related crystallographic study on a neutral species related to the title compound, see: Kaiser *et al.* (2009 ▶). For the synthesis, see: Elderfield *et al.* (1946 ▶).
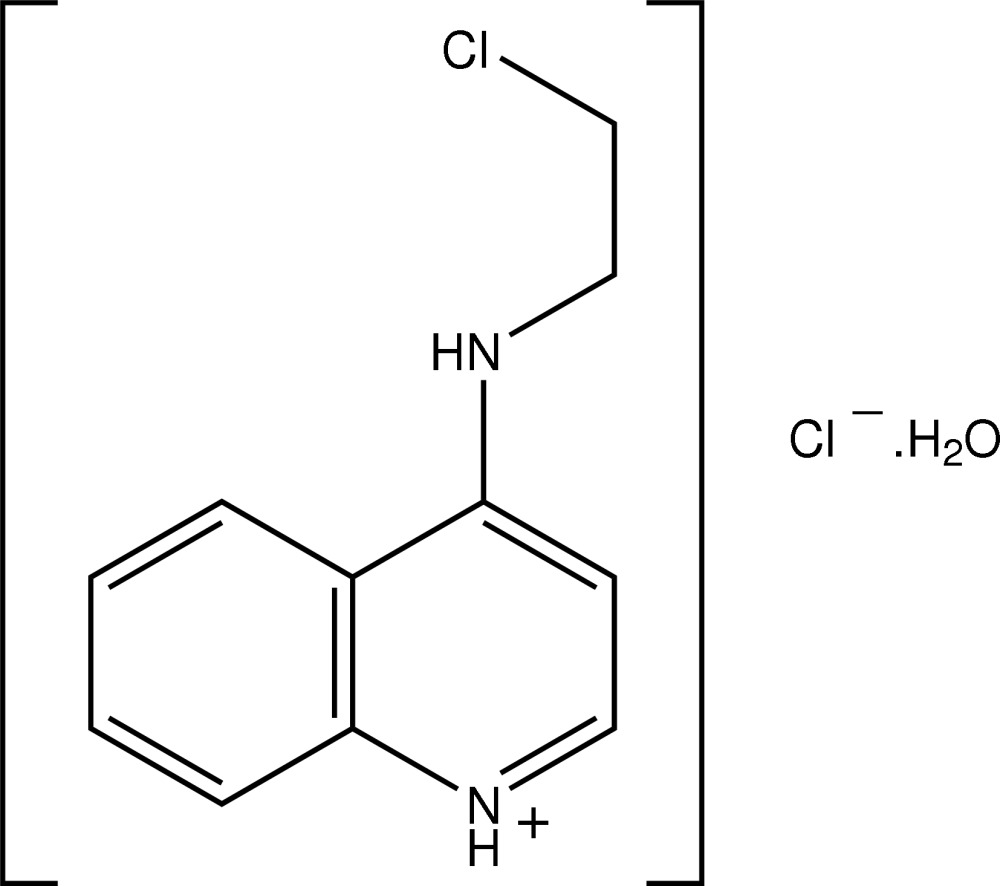


## Experimental

### 

#### Crystal data

C_11_H_12_ClN_2_^+^·Cl^−^·H_2_O
*M* *_r_* = 261.14
Orthorhombic, 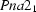

*a* = 18.7513 (7) Å
*b* = 14.1030 (5) Å
*c* = 4.606 (1) Å
*V* = 1218.1 (3) Å^3^
*Z* = 4
Mo *K*α radiation
μ = 0.51 mm^−1^
*T* = 120 K
0.46 × 0.03 × 0.03 mm

#### Data collection

Bruker–Nonius 95mm CCD camera on κ-goniostat diffractometer
Absorption correction: multi-scan (*SADABS*; Sheldrick, 2003 ▶) *T*_min_ = 0.816, *T*_max_ = 1
11264 measured reflections
2681 independent reflections
2390 reflections with *I* > 2σ(*I*)
*R*_int_ = 0.049

#### Refinement

*R*[*F* ^2^ > 2σ(*F* ^2^)] = 0.032
*wR*(*F* ^2^) = 0.065
*S* = 1.06
2681 reflections
151 parameters
4 restraints
H-atom parameters constrained
Δρ_max_ = 0.22 e Å^−3^
Δρ_min_ = −0.21 e Å^−3^
Absolute structure: Flack (1983 ▶), 1123 Friedel pairs
Flack parameter: 0.03 (6)

### 

Data collection: *COLLECT* (Hooft, 1998 ▶); cell refinement: *DENZO* (Otwinowski & Minor, 1997 ▶) and *COLLECT*; data reduction: *DENZO* and *COLLECT*; program(s) used to solve structure: *SHELXS97* (Sheldrick, 2008 ▶); program(s) used to refine structure: *SHELXL97* (Sheldrick, 2008 ▶); molecular graphics: *DIAMOND* (Brandenburg, 2006 ▶); software used to prepare material for publication: *publCIF* (Westrip, 2009 ▶).

## Supplementary Material

Crystal structure: contains datablocks global, I. DOI: 10.1107/S160053680904834X/hg2592sup1.cif

Structure factors: contains datablocks I. DOI: 10.1107/S160053680904834X/hg2592Isup2.hkl

Additional supplementary materials:  crystallographic information; 3D view; checkCIF report

## Figures and Tables

**Table 1:** Hydrogen-bond geometry (Å, °)

*D*—H⋯*A*	*D*—H	H⋯*A*	*D*⋯*A*	*D*—H⋯*A*
N1—H1⋯O1^i^	0.88	1.84	2.710 (2)	172
N2—H2⋯Cl2	0.88	2.41	3.2298 (18)	155
O1—H1w⋯Cl2^ii^	0.84	2.32	3.1288 (19)	161
O1—H2w⋯Cl2	0.84	2.29	3.1204 (19)	173
C5—H5⋯Cl2	0.95	2.82	3.730 (2)	161

Symmetry codes: (i) 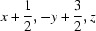; (ii) 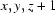.

**Fig. 1.**
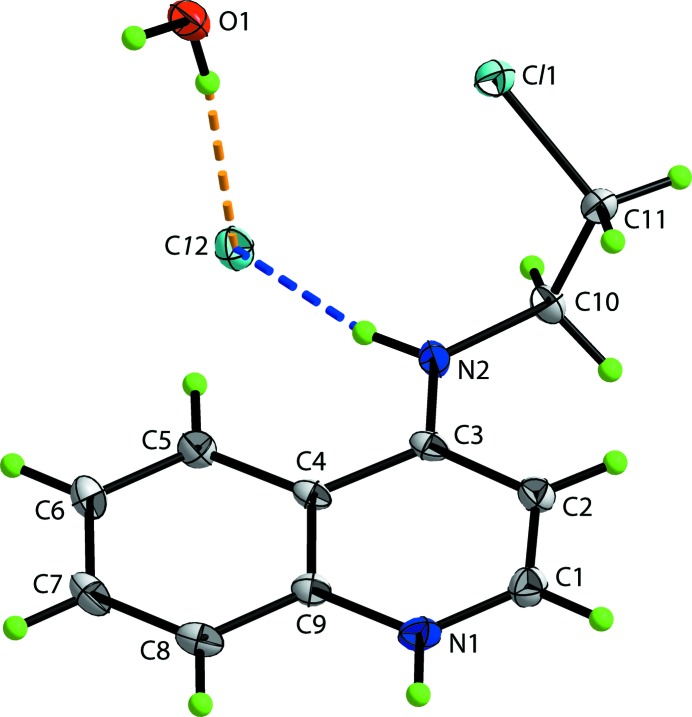
The asymmetric unit in (I) showing the atom-labelling scheme and displacement ellipsoids at the 50% probability level. Hydrogen bonding between the water molecule and Cl2 anion (orange dashed line), and between the amine-N1—H and Cl2 anion (blue dashed line) are highlighted.

**Fig. 2.**
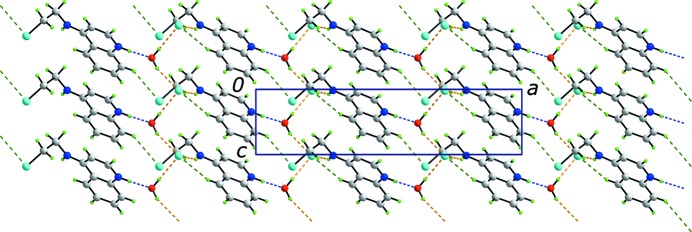
Supramolecular 2-D array in (I) in the ac plane. The N–H···O (blue), N–H···Cl and O–H···Cl (orange), and C–H···O (green) interactions are shown as dashed lines. Colour code: Cl, cyan; O, red; N, blue; C, grey; and H, green.

## supplementary crystallographic information

### Comment

The majority of drugs used against malaria, such as chloroquine (Tanenbaum & Tuffanelli, 1980), mefloquine (Palmer *et al.*, 1993), primaquine (Elslager *et al.*, 1969) and amodiaquine (Ridley, 2002) possess a quinoline ring which has been the mainstay of malaria chemotherapy for much of the past 40 years (Font *et al.*, 1997; Kaminsky & Meltzer, 1968; Musiol *et al.*, 2006; Nakamura *et al.*, 1999; Sloboda *et al.*, 1991; Warshakoon *et al.*, 2006). However, their effectiveness has been seriously eroded in recent years, mainly as a result of the development of parasite resistance (Ridley, 2002). Malaria remains one of the most important diseases of humans with over half of the world population at risk of infection. It affects mainly those living in tropical and subtropical areas with an incidence of 500 million cases per year globally (Snow *et al.*, 1999; Breman, 2001; World Health Organization, 1999). As part of our studies (de Souza *et al.*, 2005; Andrade *et al.*, 2007; da Silva *et al.*, 2003; Cunico *et al.*, 2006) of drugs for neglected diseases, various quinoline derivatives with potential antimalarial activities have been investigated. It was during this study that the the title salt hydrate, (I), was characterized.

The quinolinium core in (I), Fig. 1, is essentially planar with a RMS deviation of the 10 atoms comprising the framework being 0.027 Å, with a maximum deviation exhibited by the C3 atom [0.031 (2) Å]. The amine side-chain deviates significantly from this plane starting with the N2 atom which lies 0.082 (2) Å above the plane. Further along the side-chain, the C11 and Cl1 atoms are almost orthogonal to the quinolinium core as seen in the magnitude of the C3/N2/C10/C11 torsion angle of -80.0 (3) °. The N—H group is orientated towards the aromatic ring. These conformational features are as found in the neutral parent compound (Kaiser *et al.* (2009). The most significant difference between the geometric parameters in the neutral and protonated forms is found in the angles subtended at the N1 atom, *i.e.* this has widened considerably in (I), 121.00 (19) Å, compared with 115.3 (2) ° in the neutral form, consistent with protonation in the former.

As expected from the composition of (I), there are significant hydrogen bonding interactions operating in the crystal structure, Table 1. The quinolinium nitrogen atom forms a donor interaction to the water molecule which in turn forms two donor interactions to the Cl2 anion. The Cl2 anion accepts a hydrogen bond from the amine-H with the result that a 2-D supramolecular array is formed in the *ac* plane, Fig. 2. Additional stability to the array is provided by C–H···Cl interactions involving the Cl1 atom, Table 1. Layers stack along the *b* axis to consolidate the crystal structure.

### Experimental

A mixture of 7-chloro-*N*-(2-hydroxyethyl)quinolin-4-amine) (Kaiser *et al.*, 2009) (0.5 g, 2.2 mmol), thionyl chloride (33 ml, 45 mmol) and DMF (0.3 ml, 0.22 mol) was stirred under nitrogen at room temperature for 24 h. The resulting mixture was treated with a saturated aqueous solution of sodium bicarbonate and extracted with ethyl acetate. The combined organic phases were dried over anhydrous sodium sulfate and concentrated under reduced pressure to yield solid (I); yield: 94%.The compound was recrystallized from ethanol, m. pt.: 402–403 K. The melting point of the free base was reported to be 427 K (Elderfield *et al.*, 1946).

### Refinement

The C-bound H atoms were geometrically placed (C–H = 0.95–0.99 Å) and refined as riding with *U*_iso_(H) = 1.2*U*_eq_(C). The N-bound H atoms were located from a difference map and included in their idealized positions with N–H = 0.88 Å, and with *U*_iso_(H) = 1.2*U*_eq_(N). The water-H atoms were located from a difference map and refined with O–H = 0.840±0.001 Å and H···H = 1.39±0.01 Å, and with *U*_iso_(H) = 1.5*U*_eq_(O).

### Crystal data

**Table:**

C_11_H_12_ClN_2_^+^·Cl^−^·H_2_O	*F*(000) = 544
*M**_r_* = 261.14	*D*_x_ = 1.424 Mg m^−^^3^
Orthorhombic, *P**n**a*2_1_	Mo *K*α radiation, λ = 0.71070 Å
Hall symbol: P 2c -2n	Cell parameters from 1650 reflections
*a* = 18.7513 (7) Å	θ = 2.9–27.5°
*b* = 14.1030 (5) Å	µ = 0.51 mm^−^^1^
*c* = 4.606 (1) Å	*T* = 120 K
*V* = 1218.1 (3) Å^3^	Needle, colourless
*Z* = 4	0.46 × 0.03 × 0.03 mm

### Data collection

**Table:**

Bruker–Nonius 95mm CCD camera on κ-goniostat diffractometer	2681 independent reflections
Radiation source: Bruker-Nonius FR591 rotating anode	2390 reflections with *I* > 2σ(*I*)
graphite	*R*_int_ = 0.049
Detector resolution: 9.091 pixels mm^-1^	θ_max_ = 27.5°, θ_min_ = 3.1°
φ & ω scans	*h* = −19→24
Absorption correction: multi-scan (*SADABS*; Sheldrick, 2003)	*k* = −18→18
*T*_min_ = 0.816, *T*_max_ = 1	*l* = −5→5
11264 measured reflections	

### Refinement

**Table:**

Refinement on *F*^2^	Secondary atom site location: difference Fourier map
Least-squares matrix: full	Hydrogen site location: inferred from neighbouring sites
*R*[*F*^2^ > 2σ(*F*^2^)] = 0.032	H-atom parameters constrained
*wR*(*F*^2^) = 0.065	*w* = 1/[σ^2^(*F*_o_^2^) + (0.0173*P*)^2^ + 0.5206*P*] where *P* = (*F*_o_^2^ + 2*F*_c_^2^)/3
*S* = 1.06	(Δ/σ)_max_ = 0.001
2681 reflections	Δρ_max_ = 0.22 e Å^−^^3^
151 parameters	Δρ_min_ = −0.21 e Å^−^^3^
4 restraints	Absolute structure: Flack (1983), 1123 Friedel pairs
Primary atom site location: structure-invariant direct methods	Flack parameter: 0.03 (6)

### Special details

**Table:**

Geometry. All e.s.d.'s (except the e.s.d. in the dihedral angle between two l.s. planes) are estimated using the full covariance matrix. The cell e.s.d.'s are taken into account individually in the estimation of e.s.d.'s in distances, angles and torsion angles; correlations between e.s.d.'s in cell parameters are only used when they are defined by crystal symmetry. An approximate (isotropic) treatment of cell e.s.d.'s is used for estimating e.s.d.'s involving l.s. planes.
Refinement. Refinement of *F*^2^ against ALL reflections. The weighted *R*-factor *wR* and goodness of fit *S* are based on *F*^2^, conventional *R*-factors *R* are based on *F*, with *F* set to zero for negative *F*^2^. The threshold expression of *F*^2^ > σ(*F*^2^) is used only for calculating *R*-factors(gt) *etc*. and is not relevant to the choice of reflections for refinement. *R*-factors based on *F*^2^ are statistically about twice as large as those based on *F*, and *R*- factors based on ALL data will be even larger.

### Fractional atomic coordinates and isotropic or equivalent isotropic displacement parameters (Å^2^)

**Table:**

	*x*	*y*	*z*	*U*_iso_*/*U*_eq_	
Cl1	0.13741 (3)	0.81412 (4)	0.18205 (16)	0.02165 (12)	
Cl2	0.21007 (3)	0.53250 (3)	0.01935 (15)	0.02136 (12)	
O1	0.10851 (8)	0.59720 (11)	0.5181 (4)	0.0271 (4)	
H1W	0.1283	0.5858	0.6780	0.041*	
H2W	0.1365	0.5852	0.3805	0.041*	
N1	0.48246 (10)	0.82732 (12)	0.3467 (4)	0.0188 (4)	
H1	0.5241	0.8470	0.4104	0.023*	
N2	0.29024 (9)	0.73519 (11)	0.0155 (4)	0.0163 (3)	
H2	0.2742	0.6796	0.0728	0.020*	
C1	0.44596 (12)	0.88075 (15)	0.1575 (5)	0.0203 (5)	
H1A	0.4657	0.9397	0.0982	0.024*	
C2	0.38179 (12)	0.85382 (14)	0.0472 (5)	0.0184 (4)	
H2A	0.3572	0.8943	−0.0839	0.022*	
C3	0.35148 (11)	0.76568 (14)	0.1268 (4)	0.0158 (5)	
C4	0.39002 (11)	0.70904 (15)	0.3390 (5)	0.0152 (4)	
C5	0.36387 (12)	0.62250 (15)	0.4527 (4)	0.0179 (5)	
H5	0.3194	0.5985	0.3873	0.021*	
C6	0.40215 (12)	0.57290 (15)	0.6566 (5)	0.0208 (5)	
H6	0.3837	0.5153	0.7328	0.025*	
C7	0.46856 (13)	0.60686 (16)	0.7533 (5)	0.0230 (5)	
H7	0.4951	0.5715	0.8917	0.028*	
C8	0.49518 (11)	0.69039 (15)	0.6493 (5)	0.0192 (5)	
H8	0.5399	0.7133	0.7159	0.023*	
C9	0.45584 (11)	0.74244 (15)	0.4426 (4)	0.0158 (5)	
C10	0.24785 (13)	0.78772 (16)	−0.1954 (5)	0.0181 (5)	
H10A	0.2162	0.7429	−0.2993	0.022*	
H10B	0.2802	0.8167	−0.3402	0.022*	
C11	0.20268 (11)	0.86510 (15)	−0.0584 (4)	0.0177 (5)	
H11A	0.1783	0.9017	−0.2125	0.021*	
H11B	0.2337	0.9091	0.0515	0.021*	

### Atomic displacement parameters (Å^2^)

**Table:**

	*U*^11^	*U*^22^	*U*^33^	*U*^12^	*U*^13^	*U*^23^
Cl1	0.0166 (3)	0.0257 (3)	0.0227 (3)	−0.0021 (2)	0.0006 (2)	0.0039 (3)
Cl2	0.0218 (3)	0.0193 (2)	0.0230 (3)	−0.0009 (2)	−0.0043 (2)	0.0008 (3)
O1	0.0220 (9)	0.0335 (9)	0.0258 (8)	0.0085 (7)	0.0002 (8)	0.0060 (9)
N1	0.0138 (10)	0.0193 (10)	0.0234 (10)	−0.0035 (8)	−0.0001 (8)	−0.0019 (8)
N2	0.0164 (9)	0.0140 (8)	0.0186 (8)	0.0010 (7)	−0.0025 (8)	0.0033 (9)
C1	0.0211 (11)	0.0162 (10)	0.0235 (11)	−0.0012 (9)	0.0026 (10)	0.0002 (10)
C2	0.0187 (11)	0.0169 (10)	0.0196 (11)	0.0036 (9)	0.0018 (10)	0.0010 (10)
C3	0.0136 (11)	0.0169 (10)	0.0168 (12)	0.0041 (8)	0.0021 (8)	−0.0028 (8)
C4	0.0135 (11)	0.0158 (10)	0.0163 (11)	0.0040 (8)	0.0009 (8)	−0.0036 (8)
C5	0.0172 (12)	0.0158 (10)	0.0206 (12)	−0.0003 (9)	−0.0029 (8)	−0.0015 (9)
C6	0.0235 (12)	0.0156 (10)	0.0235 (11)	0.0031 (9)	−0.0009 (11)	0.0002 (10)
C7	0.0237 (13)	0.0235 (12)	0.0217 (13)	0.0108 (10)	−0.0031 (9)	−0.0017 (9)
C8	0.0139 (11)	0.0241 (11)	0.0196 (12)	0.0031 (9)	−0.0012 (10)	−0.0062 (9)
C9	0.0160 (11)	0.0162 (10)	0.0151 (11)	0.0020 (9)	0.0022 (8)	−0.0032 (8)
C10	0.0175 (12)	0.0193 (11)	0.0175 (11)	0.0016 (9)	−0.0032 (9)	0.0011 (8)
C11	0.0155 (12)	0.0186 (11)	0.0192 (12)	−0.0011 (9)	0.0009 (8)	0.0029 (8)

### Geometric parameters (Å, °)

**Table:**

Cl1—C11	1.800 (2)	C4—C5	1.416 (3)
O1—H1W	0.8400	C5—C6	1.374 (3)
O1—H2W	0.8401	C5—H5	0.9500
N1—C1	1.340 (3)	C6—C7	1.407 (3)
N1—C9	1.370 (3)	C6—H6	0.9500
N1—H1	0.8800	C7—C8	1.366 (3)
N2—C3	1.329 (3)	C7—H7	0.9500
N2—C10	1.457 (3)	C8—C9	1.411 (3)
N2—H2	0.8800	C8—H8	0.9500
C1—C2	1.360 (3)	C10—C11	1.519 (3)
C1—H1A	0.9500	C10—H10A	0.9900
C2—C3	1.415 (3)	C10—H10B	0.9900
C2—H2A	0.9500	C11—H11A	0.9900
C3—C4	1.454 (3)	C11—H11B	0.9900
C4—C9	1.405 (3)		
			
H1W—O1—H2W	110.3	C5—C6—H6	119.8
C1—N1—C9	121.00 (19)	C7—C6—H6	119.8
C1—N1—H1	119.5	C8—C7—C6	120.4 (2)
C9—N1—H1	119.5	C8—C7—H7	119.8
C3—N2—C10	124.33 (17)	C6—C7—H7	119.8
C3—N2—H2	117.8	C7—C8—C9	119.6 (2)
C10—N2—H2	117.8	C7—C8—H8	120.2
N1—C1—C2	122.5 (2)	C9—C8—H8	120.2
N1—C1—H1A	118.7	N1—C9—C4	120.24 (19)
C2—C1—H1A	118.7	N1—C9—C8	118.81 (19)
C1—C2—C3	120.2 (2)	C4—C9—C8	120.95 (19)
C1—C2—H2A	119.9	N2—C10—C11	113.12 (18)
C3—C2—H2A	119.9	N2—C10—H10A	109.0
N2—C3—C2	122.08 (19)	C11—C10—H10A	109.0
N2—C3—C4	120.73 (18)	N2—C10—H10B	109.0
C2—C3—C4	117.2 (2)	C11—C10—H10B	109.0
C9—C4—C5	117.89 (19)	H10A—C10—H10B	107.8
C9—C4—C3	118.74 (19)	C10—C11—Cl1	110.35 (15)
C5—C4—C3	123.36 (19)	C10—C11—H11A	109.6
C6—C5—C4	120.7 (2)	Cl1—C11—H11A	109.6
C6—C5—H5	119.6	C10—C11—H11B	109.6
C4—C5—H5	119.6	Cl1—C11—H11B	109.6
C5—C6—C7	120.4 (2)	H11A—C11—H11B	108.1
			
C9—N1—C1—C2	−1.0 (3)	C5—C6—C7—C8	1.2 (3)
N1—C1—C2—C3	−1.1 (4)	C6—C7—C8—C9	−0.4 (3)
C10—N2—C3—C2	−0.3 (3)	C1—N1—C9—C4	1.2 (3)
C10—N2—C3—C4	179.32 (19)	C1—N1—C9—C8	−177.8 (2)
C1—C2—C3—N2	−177.4 (2)	C5—C4—C9—N1	−177.87 (18)
C1—C2—C3—C4	2.9 (3)	C3—C4—C9—N1	0.7 (3)
N2—C3—C4—C9	177.64 (19)	C5—C4—C9—C8	1.2 (3)
C2—C3—C4—C9	−2.7 (3)	C3—C4—C9—C8	179.73 (18)
N2—C3—C4—C5	−3.9 (3)	C7—C8—C9—N1	178.23 (19)
C2—C3—C4—C5	175.8 (2)	C7—C8—C9—C4	−0.8 (3)
C9—C4—C5—C6	−0.4 (3)	C3—N2—C10—C11	−80.0 (3)
C3—C4—C5—C6	−178.9 (2)	N2—C10—C11—Cl1	−63.9 (2)
C4—C5—C6—C7	−0.8 (3)		

### Hydrogen-bond geometry (Å, °)

**Table:**

*D*—H···*A*	*D*—H	H···*A*	*D*···*A*	*D*—H···*A*
N1—H1···O1^i^	0.88	1.84	2.710 (2)	172
N2—H2···Cl2	0.88	2.41	3.2298 (18)	155
O1—H1w···Cl2^ii^	0.84	2.32	3.1288 (19)	161
O1—H2w···Cl2	0.84	2.29	3.1204 (19)	173
C5—H5···Cl2	0.95	2.82	3.730 (2)	161

Symmetry codes: (i) *x*+1/2, −*y*+3/2, *z*; (ii) *x*, *y*, *z*+1.
